# Electrochemical Oxidation of Ti15Mo Alloy—The Impact of Anodization Parameters on Surface Morphology of Nanostructured Oxide Layers

**DOI:** 10.3390/nano11010068

**Published:** 2020-12-30

**Authors:** Magdalena Jarosz, Leszek Zaraska, Marcin Kozieł, Wojciech Simka, Grzegorz D. Sulka

**Affiliations:** 1 Faculty of Chemistry, Jagiellonian University, Gronostajowa 2, 30387 Krakow, Poland; marcin.koziel@uj.edu.pl (M.K.); sulka@chemia.uj.edu.pl (G.D.S.); 2 Faculty of Chemistry, Silesian University of Technology, B. Krzywoustego 6, 44100 Gliwice, Poland; Wojciech.Simka@polsl.pl

**Keywords:** nanostructured oxide morphology, electrochemical oxidation, Ti15Mo alloy, anodization parameters

## Abstract

It is well-known that the structure and composition of the material plays an important role in the processes occurring at the surface. In this paper, a surface morphology of nanostructured oxide layers electrochemically grown on Ti15Mo, tuned by applying different anodization parameters, was investigated in detail. The one-step anodization of Ti15Mo alloy was performed at room temperature in an ethylene glycol-based electrolyte containing 0.11 M NH_4_F and 1.11 M H_2_O. Different anodization times (ranging from 5 to 60 min) and applied potentials (40–100 V) were tested, and the surface morphology, elemental content, and crystalline structure were monitored by scanning electron microscopy (SEM), energy dispersive X-ray spectrometry (EDS), and X-ray diffractometry (XRD), respectively. The results showed that contrary to the multistep anodization of titanium foil, the surface morphology of anodic oxide obtained via the one-step process contains the nanoporous outer layer covering the nanotubular structure. What is more, the pore diameter (D_p_) and interpore distance (D_int_) of such layers exhibit different trends than those observed for anodization of pure titanium. In particular, at a certain potential range, a decrease in both D_p_ and D_int_ with increasing potential was observed. However, independently on the used anodization conditions, the elemental content of oxide layers remained similar, showing the amount of molybdenum at c.a. 15 wt.%. Finally, the amorphous nature of as-anodized layers was confirmed, and their optical band-gap was determined from the diffuse reflectance UV–Vis spectra. It was found that E_g_ is tunable to some extent by changing the anodizing potential. However, further thermal treatment in air at 400 °C resulted in the anatase phase formation that was accompanied by a significant E_g_ reduction. Therefore, we believe that the presented results will greatly contribute to the understanding of anodic formation of nanostructured functional oxide layers with tunable properties that can be applied in various fields.

## 1. Introduction

Different titanium alloys gained an increasing interest in the last two decades, gradually replacing commercially pure Ti (CP-Ti). This was mainly due to their wide range of applications, starting from the aerospace industry [1,2] up to medicine [3,4,5], which is usually a result of better properties of Ti-based alloys. Depending on the additive element used to produce the alloy, they are divided into six groups, i.e., α and near α, (α + β), β and near β, and finally shape memory Ti-based alloys [6]. Among them, alloys with β-phase stabilizers (e.g., Cr, Nb, V, Mo) seem to be particularly interesting since they are characterized by higher strength, better corrosion resistance, and biocompatibility, when compared to pure Ti and other types of Ti-based alloys.

To further widen the applicability of such materials, surface modification of titanium and its alloys has been developed. One of the most widely used methods is electrochemical oxidation of metals, known as anodization resulting in the formation of the oxide film on a particular metal or alloy [7,8]. It has been intensively studied with respect to aluminum [9], titanium [8], tungsten [10], tin [11], and zinc [12] in the past few decades. However, it is often used to modify different alloys’ surfaces to obtain mixed oxides offering many promising properties [5,13]. The advantages of anodization are its low costs, simplicity, and, above all, the possibility to easily form oxide films with various morphologies. Depending on oxidation parameters, such as applied potential, time of the process, electrolyte, or temperature, it is possible to obtain barrier-type oxides, nanoporous layers, or nanotubes (NTs) [7,9].

As already mentioned, β-phase alloys may be an interesting alternative for applicability, where some of the most commonly investigated are titanium-molybdenum alloys with different Mo contents [14,15,16,17,18,19,20,21]. The synthesis of an oxide layer on such surfaces may be beneficial from the implantology point of view, since it is well-known that nanotopography facilitates adhesion and proliferation of osteoblast cells [22,23], whereas molybdenum oxides may have antibacterial properties [24,25]. In addition, the possibility of the direct formation of nanostructured Mo-containing TiO_2_ via simple one-step anodic oxidation seems to be especially encouraging, since it is widely known that such kind of materials can offer enhanced photoelectrochemical [26] and photocatalytic [27] properties under visible light.

Although anodization of such alloys is being investigated for several years now, little is known on the influence of anodization parameters on the morphology of oxide layers and their properties (see Table 1).

As shown, the main focus of the previous research devoted to anodization of TiMo alloys was put on the effect of Mo content in the alloy on the oxide layer morphology. It was found that by simply changing the amount of β-type additive in the alloy, various types of oxide layers can be synthesized (i.e., nanotubes, nanopores, or barrier-type layers). It is worth mentioning that similar results were also obtained for other Ti-based alloys, e.g., TiNb [35,36], TiZr [37,38], and TiTa [39]. Different nanostructures may also be achieved by changing either the electrolyte composition or other anodization conditions [3,7,18,40]. As it was proven for the anodization of different metals, the applied potential influences the diameter of nanotubes. For TiMo layers, tubes with a diameter ranging from 15 up to 120 nm were obtained, depending on the applied potential, and anodization time used for the process [28,32]. Different types of electrolytes, both inorganic [20,30] and organic [17,18,28,29,31,32,33,34], were tested, and in most cases, nanotubular TiO_2_ layers were received. Some groups also reported a usage of the ‘aged’ electrolyte as an alternative for the freshly prepared one [28,34]. However, it is not only a long-term and expensive procedure, but also may result in a significant deterioration of TiO_2_ NT properties [41].

Interestingly, for some of the TiMo alloys, a nanoporous layer on the top of nanotubes was reported [28,29,31,34]. It was mostly found for alloys with a small amount of molybdenum (i.e., less than 10 wt.%), despite the applied anodization conditions. What is more, Agarwal et al. [37] reported a porous oxide structure formed on layers with a significantly higher Mo content, namely 20 and 35 wt.%, and confirmed the alloy composition is an important parameter influencing the growth rate of the nanostructured film. However, the nature of the formation of porous or tubular layers on TiMo alloys seems to be more complex, and further thorough studies are required. Moreover, a detailed analysis of the morphology of the outer oxide film, i.e., the layer formed during the initial stages of anodization under different conditions, is still almost completely lacking.

Therefore, the aim of this work is to show how the anodization parameters, i.e., duration and applied potential, influence the outer oxide layer formed on the Ti15Mo alloy. To the best of our knowledge, this is the first time that such extensive research on anodization of the Ti15Mo alloy has been conducted. The in-depth research on this subject is especially important, since the morphology of the surface of Ti-based materials play a crucial role in their application, e.g., in medicine, where it is responsible for the initial adherence of cells. Moreover, it is also important for the processes occurring in photocatalysis or photoelectrochemistry, whereby tuning the morphology of the semiconducting material, the efficiency of the processes may be significantly increased. This research may provide some important information for designing nanomaterials for specific applications.

## 2. Materials and Methods

### 2.1. Electrochemical Oxidation of Ti15Mo Alloy

The disc samples of Ti15Mo alloy (5 mm thick) were provided by BIMO Metals (Wroclaw, Poland). Prior to electrochemical oxidation, samples were polished according to the procedure established previously [42]. Briefly, the alloy surface was electrochemically polished under a constant current density of 50 A dm^−2^ for 3 min in the aqueous solution containing 1.1 M oxalic acid, 2.7 M ammonium fluoride, 5.3 M ethylene glycol, and 10 M sulfuric acid [42]. Before oxidation, samples were insulated with an acid resistant paint, and the working area of all samples was always equal to 0.79 cm^2^.

Electrochemical oxidation (anodization) of samples was carried out in the ethylene glycol-based electrolyte containing 0.11 M NH_4_F and 1.11 M H_2_O. The one-step process was carried out at a constant temperature (20 °C) and constant stirring speed (150 rpm). Anodization was performed in a two-electrode cell, where the alloy sample and Ti plate (2 × 2 cm) were used as an anode and cathode, respectively. The anodization conditions (time and potential (U)) are shown in Table 2.

During anodization, current vs. time plots were recorded for all samples. After the process, samples were gently rinsed with distilled water and dried in air.

The structural and morphological characterizations of anodic layers formed on the alloy surface was performed by using a field emission scanning electron microscope (FE-SEM, Hitachi S-4700, Krefeld, Germany). The distribution of elements in the oxide layer was determined with an energy-dispersive X-ray spectrometer (EDS) coupled with SEM. EDS elemental maps were recorded for each sample at the magnification of 2.0 k. Pore diameter (D_p_) and interpore distance (D_int_) were calculated for each sample using the scanning probe image processor WSxM 5.0 Develop 9.3 [43].

### 2.2. Crystalline Structure of Anodized Oxide Layers

Some of the anodized samples were subjected to the annealing process to change its amorphous structure into anatase. Based on our previous work on heat treatment of anodized titanium foil [44], we chose the annealing parameters to be as follows: annealing temperature 400 °C, heating rate 2° min^−1^, annealing time 2 h. Both, as-prepared and annealed samples were characterized using the X-ray diffraction method. XRD patterns were registered in the Bragg–Brentano geometry using the PANalytical X’Pert PRO MPD (Malvern, UK) diffractometer in the range of 5–80° 2θ. A tube with a copper anode was used as an X-ray source (radiation Cu Kα_12_, λ_avg_ = 1.54178 Å split into Kα1 and Kα2 with 2:1 ratio). The results were analyzed by means of PDF-4+ database [45] using X’Pert HighScore software (version 4.9, Malvern Panalytical, Malvern, UK) [46]. Moreover, similarly to the as-received samples, annealed oxide layers were characterized using SEM and EDS.

### 2.3. UV–Vis Characterization

UV–Vis reflectance spectra were recorded using the Lambda 750S spectrophotometer (PerkinElmer, Waltham, MA, USA) equipped with an integrating sphere module. The diffuse reflectance of all samples was recorded in the range of 250–800 nm with a step size of 2 nm. The Spectralon^®^ SRS-99-010 diffuse reflectance standard was used as a reference. Data processing was performed using the PerkinElmer UV WinLab Data Processor and Viewer.

## 3. Results and Discussion

**Figure 1:**
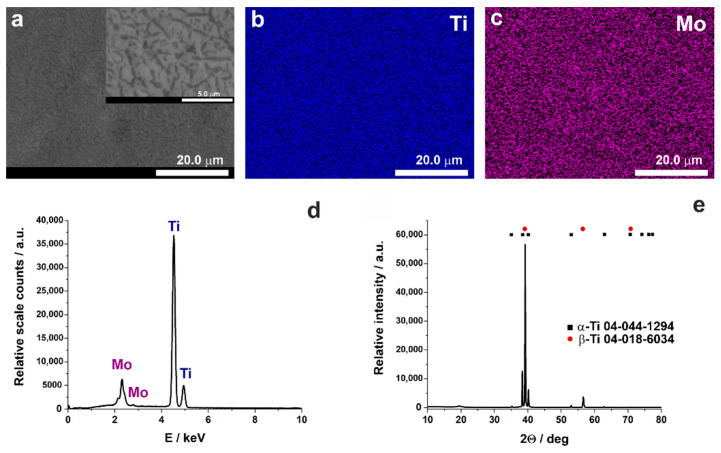
FE-SEM image (low and high (inset) magnification) (**a**), EDS elemental maps (**b**,**c**), EDS spectrum (**d**), and XRD pattern (**e**) of as-received Ti15Mo substrate prior to anodization process.

At first, a detailed characterization of the starting material was performed, and the results are shown in Figure 1.

FE-SEM image reveals that the surface of the material after polishing is relatively smooth with some distinct domains suggesting the coexistence of both β and α phases, that was also confirmed by XRD measurements (see Figure 1e). However, it should be emphasized that, as expected, the β phase is predominant for this alloy, which is consistent with previous reports (e.g., [47]). Moreover, EDS elemental maps (Figure 1b,c) show a uniform distribution of both elements (Ti and Mo). The alloy composition was verified to be 75 wt.% and 15 wt.% for titanium and molybdenum, respectively. Such a substrate was then anodized in the ethylene glycol-based solution containing fluoride ions and a small amount of water. 

As shown in Table 1, there is very limited data on the electrochemical oxidation of the Ti15Mo alloy. Most groups focused on the influence of molybdenum content on the morphology of obtained oxide layers, though little is known of the impact of anodization parameters on the morphology of anodic films. That is why we decided to thoroughly investigate how time and the anodization potential influence the pore diameter and interpore distance in the anodic titania layers.

**Figure 2:**
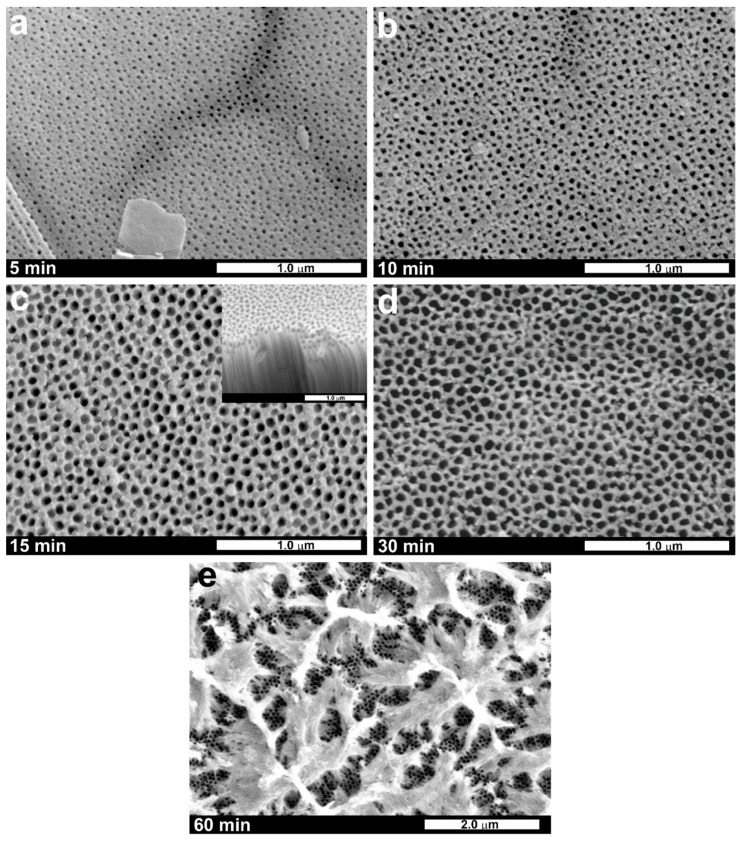
SEM microphotographs of the Ti15Mo alloy anodized in the ethylene glycol-based electrolyte under 40 V for different periods ((**a**)—5 min, (**b**)—10 min, (**c**)—15 min, (**d**)—30 min, and (**e**)—60 min). The inset in (**c**) shows an exemplary cross-section of the obtained oxide layer. Magnification of (**a**–**d**) was 50.0 k; (**e**) was 20.0 k.

Considering the Authors’ experience in anodization of pure titanium, the initial conditions were determined based on previous papers [8,48], i.e., the anodization potential and temperature were fixed at 40 V and 20 °C, respectively. Different anodization durations were chosen, starting from 5 min up to 1 h. The SEM images of the surface of Ti15Mo anodized for different durations are shown in Figure 2.

**Figure 3:**
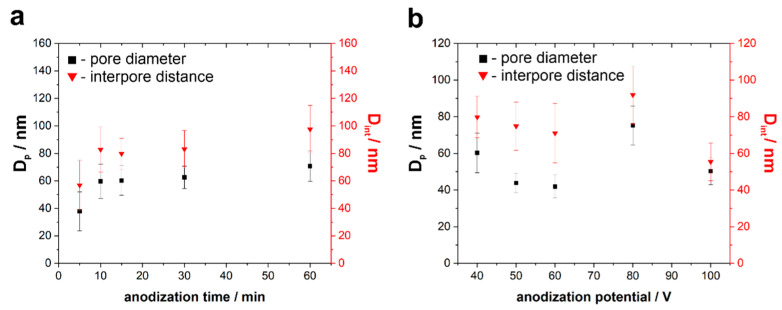
Pore diameter (D_p_) and interpore distance (D_int_) as a function of anodizing time (**a**) and anodizing potential (**b**). The electrochemical oxidation of Ti15Mo alloy was conducted in the ethylene glycol-based electrolyte at 20 °C. The error bars represent the standard deviation from at least 50 measurements from three separate samples anodized at the same conditions.

As can be seen, the samples anodized for 5–30 min exhibit a uniform nanoporous morphology (Figure 2a–d). In addition, the average pore diameter increased from c.a. 40 nm to over 60 nm when the anodizing duration was extended from 5 to 30 min, respectively (see Figure 3a). This phenomenon, also observed for other anodic metal oxides [28,49,50], can be explained in terms of compact oxide film formation during the initial stage of anodization and its gradual transformation into a porous layer. At first, due to the effective etching of passive film by fluoride ions, nascent pores start to grow randomly. Further, anodic oxidation results in the gradual pore widening due to the prolonged field-assisted chemical etching of the anodic layer. Moreover, merging of some initially formed pores occurs during the pore rearrangement [48], which is reflected in a significant increase in the pore to pore distance from c.a. 55 nm to c.a. 85 nm (Figure 3a) when the anodization is prolonged from 5 to 10 min, respectively.

It should also be mentioned that, since a one-step anodization process is applied, all the layers are characterized by randomly distributed pores. According to the calculations, we observe a non-uniform diameter and spacing (as indicated by a high standard deviation for both D_p_ and D_int_; see Figure 3a). Nevertheless, after 10 min of anodization at 40 V, the surface of the oxide layer seems to reach the ultimate morphology (no significant changes in D_p_ and D_int_ can be seen, even when anodization was prolonged to 30 min). As shown in Figure 2e, a further increase of the anodizing time to 60 min results in the formation of a fibrous-like structure with a nanoporous layer underneath. This is mainly due to the enhanced chemical dissolution of the anodic film’s surface by F^−^ ions (much longer contact of the initially formed oxide with the electrolyte). However, both the pore diameter and interpore distance within the inner part of the film are close to those observed on the surface of samples anodized for 15 and 30 min (Figure 3a). 

What is more, a cross-sectional view of the oxide layer (inset in Figure 2c) reveals the existence of the nanoporous layer covering slightly larger nanotubes, similar to those observed by other authors on anodized TiMo alloys with various compositions, such as Ti6Mo [32], Ti7Mo [28,34], and Ti10Mo [31]. The thickness of this initially formed layer may even exceed 100 nm and depends on the alloy composition and anodization conditions [28]. Contrary to this, no initiation layers, or much thinner initiation layers, were typically observed for anodic films formed by anodization of pure Ti under the same conditions [34]. Moreover, as proven by Agarwal et al. [34], the preferential formation of the nanoporous layer occurs during anodization of TiMo alloys with a higher Mo content (i.e., Ti20Mo and Ti35Mo). In comparison, the nanotubular morphology of the inner part of the film is observed for those with a lower content of the alloying element. Such a dual oxide structure was also observed by Chen et al. [31] for TiTa and TiMo alloys with 20 and 10 wt.% of the additive, respectively. Therefore, its existence for the Ti15Mo alloy is well justified and may be beneficial from the application point of view, especially when the material surface plays a crucial role (e.g., in photocatalysis or implantology).

**Figure 4:**
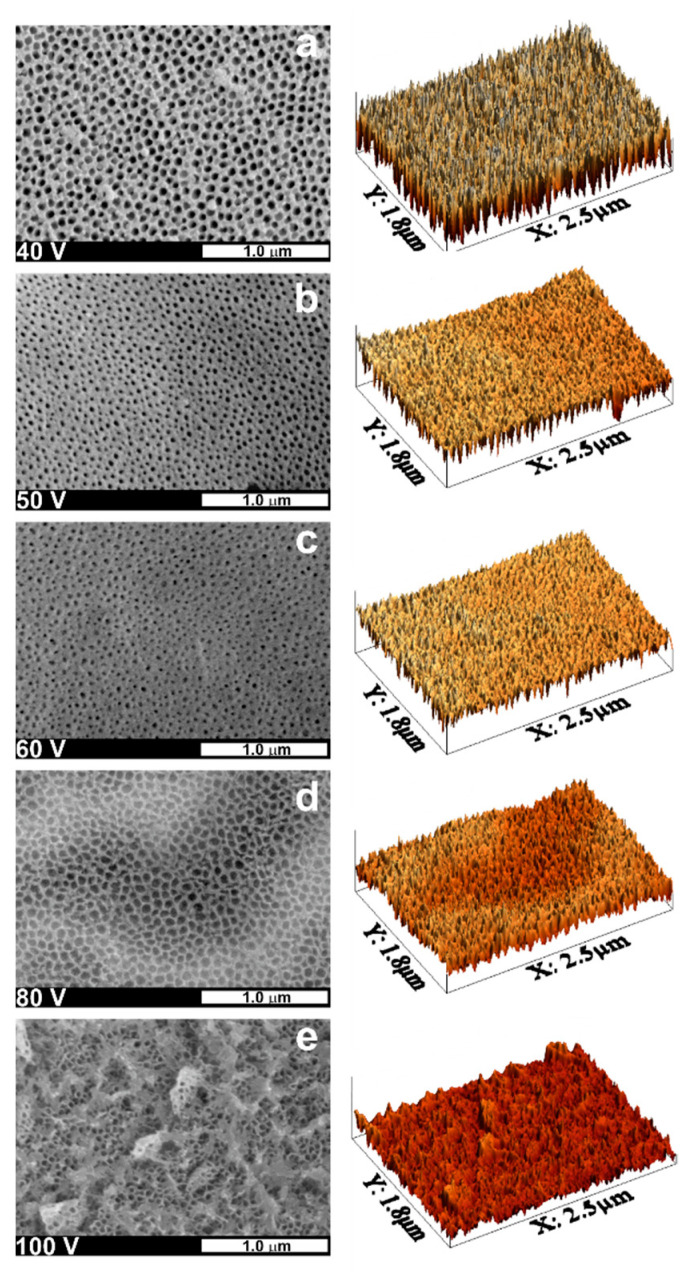
FE-SEM microphotographs of the surface of Ti15Mo alloy samples anodized in the ethylene glycol-based solution for 15 min at different applied potentials (i.e., (**a**)—40 V, (**b**)—50 V, (**c**)—60 V, (**d**)—80 V, and (**e**)—100 V), together with 3D representatives of the respective FE-SEM images. The magnification of the SEM images was 50.0 k.

Considering the oxide layer parameters and the process economy, we chose 15 min as the optimal anodization time for further experiments. Therefore, in the next step, a series of 15 min anodizations at different potentials were performed. In Figure 4, FE-SEM images of received oxide layers, along with their 3D representatives, are shown.

As seen, for the samples anodized at 40, 50, and 60 V, the average pore diameter gradually decreases when increasing the anodizing potential (see also Figure 3b). Such kind of dependence is seemingly different from those observed by us and other authors for anodic layers formed on Ti [7,8,36] and many other anodic oxides [47,48,49], where typically D_p_ increases linearly when increasing the anodizing potential. Moreover, strongly linear relationships between D_p_ and U were found by different authors for anodic films formed on Ti6Mo [32], Ti7Mo [28], and Ti15Mo [32] alloys within the same potential ranges. In addition, Oliveira et al. [32] proved that the pore diameter is almost independent of the alloy composition. However, in both previous works, different electrolytes were used, and more importantly, anodizing durations were much longer (from 3 to 6 h). This indicates that in the present case, the observed outer part of the samples is still an initial layer formed at the early stages of the anodization process. It is also known that the higher the anodizing potential, the thicker barrier film is formed on the surface of metal subjected to anodic oxidation [51,52]. Therefore, although the field-assisted dissolution of oxide is enhanced at higher potentials, generation of wider pores within the initially formed film is more difficult, so, in consequence, layers with narrower outer pores are formed. 

**Figure 5:**
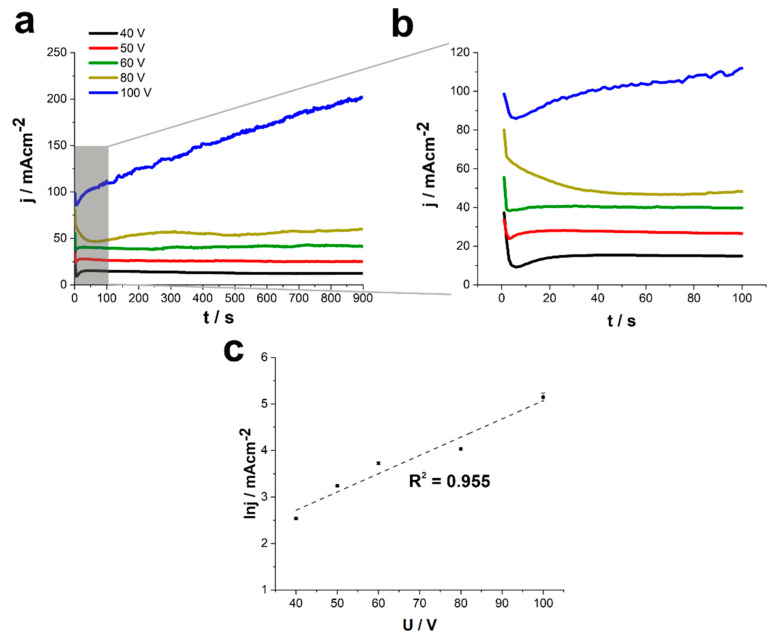
(**a**) Current density vs. time curves recorded for the anodization of Ti15Mo alloy in the ethylene glycol-based electrolyte at different potentials for 15 min. (**b**) Close-up for the initial 100 s of the process. (**c**) The natural logarithm of the steady-state current value vs. anodization potential with a linear fit.

As shown in Figure 5a,b, for processes carried out between 40 and 60 V, the current density vs. time curves exhibit a typical shape observed for anodization of different metals resulting in the formation of the porous film [7,8]. In particular, at the beginning of the process, a sudden drop in the current density related to the passive oxide layer formation, is observed. Then, a field-assisted dissolution of the oxide layer occurs, leading to the pore formation, which is represented by an increase in the recorded current density. Finally, as the current steady-state is reached, the porous anodic oxide layer grows. Moreover, the higher the anodizing potential, the shorter time required to reach the local minimum of the current density which means that the transformation of the passive layer into the porous film occurs earlier. Similar trends were observed for the anodization of pure Ti in the same electrolyte [48].

Nevertheless, it should be remembered that the growth of porous oxide occurs at the metal/oxide interface, i.e., the anodic film with the ultimate morphology (pore to pore spacing) is generated within the inner part of the layer (earlier at higher potentials), while the initiation layer (thicker when a higher potential is applied during anodization) exposed to the electrolyte is gradually etched with time prolonging. 

Another important topographic parameter that is influenced by the anodization potential is an interpore distance (D_int_). According to our best knowledge, no detailed analysis of such effect has been reported so far in the literature for anodic oxides formed on TiMo alloys. As seen in Figure 3b, a slight decrease in D_int_ with increasing the anodizing potential was also observed for samples anodized at potentials between 40 and 60 V. Contrary to that, when pure Ti was anodized under the same conditions in the same electrolyte, D_int_ of as-formed porous TiO_2_ was found to be proportional to the applied potential with a proportionality constant of c.a. 2.1 nm V^−1^ [8]. However, again, that dependence was observed for the layers obtained by the three-step anodization, i.e., pore nucleation for the final oxide layer starts in the periodic concave array formed during the initial steps [8]. In the case of the one-step process, the substrate surface is much smoother and less organized, and thus, the analyzed morphology is the morphology of the initial layer of the oxide growth, hence the observed differences in the pore diameter and interpore distance dependencies.

Surprisingly, the anodic film obtained at the potential of 80 V is characterized by significantly larger channels (c.a. 70 nm) and pore to pore distance (>90 nm). This phenomenon can be attributed to several factors, including more efficient oxide etching caused by the higher electric field, as well as a different mechanism of the oxide formation (reflected in the shape of the current density vs. time curve; see Figure 5a,b). For instance, at such a high potential, not only are much higher oxide growth rates observed, but also oxygen evolution can meaningfully affect the oxide morphology [53,54].

As shown in Figure 4e, the oxide layer grown at the potential of 100 V also exhibits the flaky morphology of the surface with a well-defined porous interior. At such a high potential, a field-assisted oxide dissolution plays a key role in forming this kind of morphology. Moreover, much higher current densities can result in a significant rise of temperature that also facilitates the oxide etching by F^−^ ions from the electrolyte. Finally, the rough shape of current density vs. time curves suggests that the oxide formation is accompanied by vigorous oxygen evolution at the anode surface.

In general, a linear relationship between the natural logarithm of a steady-state current density and the applied potential was observed (Figure 5c) that suggests that the process kinetics is limited by the migration of ions through the barrier layer, as found for TiO_2_ formed by anodization of pure Ti under the same conditions [8,50]. The observed deviations from the linearity can be caused by various factors including substrate composition (simultaneous oxidation of both metallic components occurring at different rates) and more vigorous oxygen evolution at the anode. Moreover, as seen in Figure 5c, when 100 V was applied as anodizing potential, no current stabilization was observed, which can be attributed to the continuous increase of electronic current responsible for the oxygen evolution. Therefore, this interesting issue requires further in-depth studies, which are planned for the near future.

**Figure 6:**
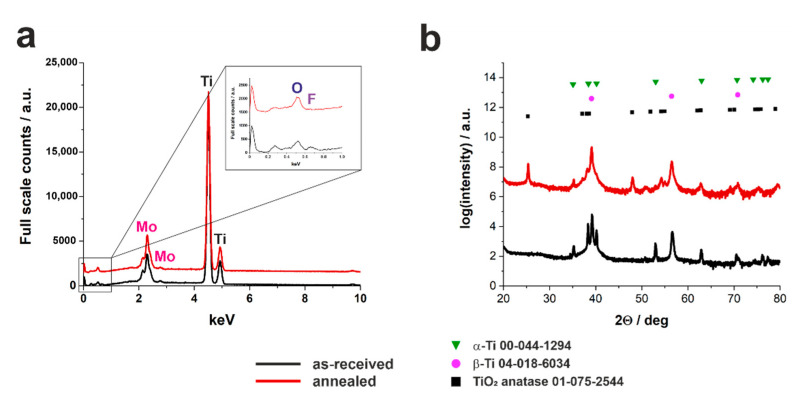
Exemplary EDS spectra (**a**) and XRD patterns (**b**) of the as-received (black lines) and annealed (red lines) Ti15Mo samples anodized at the constant potential (40 V in this case) for 15 min in the ethylene glycol-based electrolyte. The inset in (**a**) shows the magnification of the initial spectra region with the indication of oxygen and fluorine.

Typically, as-received anodic TiO_2_ layers fabricated on pure Ti and its alloys are amorphous [20,26,44]. Since for several applications, including photocatalysis and photoelectrochemical water splitting, the crystalline form of TiO_2_ is mandatory, based on our previous research [44], we have annealed the anodized samples at 400 °C. The elemental analyses of non-annealed and annealed oxide layers (Figure 6a) show prominent peaks from Ti and Mo on both types of samples. Based on the SEM analysis (data not shown), the surface morphology did not change after heat-treatment process, which is in good agreement with studies for the TiO_2_ on pure Ti [44]. The weight percentage of molybdenum in as-received and annealed oxide layers, calculated from the EDS spectra, were between 13 and 16.2 wt.%, independently on the applied potential and annealing process, and no direct correlation between the Mo content and anodizing conditions was found. Moreover, the ratios of Mo to Ti within the anodic films were found to be higher when compared to those found for the Ti15Mo substrate suggesting the preferential oxidation of Mo in the studied electrolyte.

As expected, a noticeable peak from fluorine was observed in the spectrum for as-received samples that confirms the incorporation of F^−^ ions from the electrolyte into the anodic film [40,44]. On the contrary, after thermal treatment no fluorine was found in the oxide layers, which is in perfect agreement with the results obtained for anodization of pure Ti substrate in the same electrolyte [44]. A more detailed analysis of the crystalline structure of layers showed that the as-received layers are amorphous, while after the annealing process, the anatase phase is evident in the XRD pattern (Figure 6b). As can be seen, no apparent maxima that can be attributed exclusively to any form of molybdenum oxides are visible in the XRD patterns. However, based on some previous reports regarding oxidation processes of Ti15Mo alloy, the presence of MoO_3_ in the anodic film cannot be excluded [34,42].

**Figure 7:**
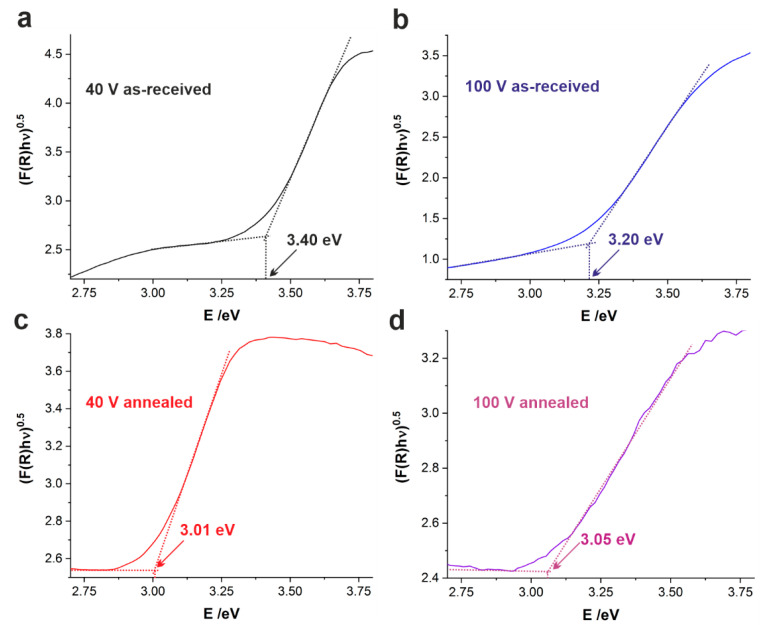
(F(R) hv)^0.5^ vs. hv plots (Tauc plots) constructed from the UV–Vis reflectance spectra of samples anodized at 40 V (**a**,**c**) and 100 V (**b**,**d**) before (**a**,**b**) and after (**c**,**d**) thermal treatment in air at 400 °C for 2 h.

Although some authors reported photocatalytic [34] and photoelectrochemical [29] applications of porous anodic films formed on TiMo alloys, no detailed examination of the effect of the applied potential on the optical band gap (E_g_) of anodically generated semiconductors can be found in the literature. To fill this void, diffuse reflectance spectra (DRS) were recorded in the UV–Vis spectral region (for example, see Figure S1) for the samples anodized at all studied potentials (both as-anodized and annealed) and converted to the Kubelka–Munk function. The optical band gap values were then determined using Tauc plots, as shown in Figure 7 (indirect nature of E_g_ was assumed).

As seen in Table 3, in general, raising the anodizing potential from 40 to 60 V results in a significant band gap narrowing, however further increase of U up to 100 V did not cause noticeable changes in E_g_ values. It can be supposed that the higher the electric field, the more ordered (but still poorly crystalline) material is formed, resulting in band gap narrowing. Although it is known that the presence of Mo within TiO_2_ nanotubes is also the reason for the narrower band gaps when compared to pure TiO_2_ [27], the Mo content in the as-synthesized anodic films was found to be independent of the applied potential (see above). Therefore, this must not be the main reason for the observed trend. The detailed explanation of this phenomenon is still not certain and requires some more in-depth studies. 

Moreover, the samples subjected to thermal treatment in air at 400 °C exhibited significantly narrower band gaps than their as-received counterparts. Since it was reported that the presence of fluoride ions within nanotubes does not affect the E_g_ of the material [54], the band gap narrowing after the thermal treatment should be attributed to the conversion of an amorphous matrix to crystalline anatase rather than changes in the composition of the anodic film (see above). Moreover, almost identical E_g_ values were observed for all annealed samples independently of the applied potential. It should be emphasized that E_g_ values of annealed layers are much lower than those observed for pure anodic TiO_2_ generated under the same conditions [44], which is consistent with some previous findings showing that the doping of titania nanotubes with Mo results in a significant narrowing of E_g_ [26,27].

## 4. Conclusions

The surface morphology of materials, especially those medically-relevant and applied in photocatalysis/photoelectrochemistry, plays a crucial role in their performance, and may impact their properties. Therefore, a detailed characterization of the oxide layers obtained during one-step electrochemical oxidation of the Ti15Mo alloy under different conditions was performed for the first time. The results showed that the obtained layers consist of the outer nanoporous layer covering nanotubes. The pore diameter of such layers reaches its maximum after 10 min of the anodization process (c.a. 60 nm and 80 nm for D_p_ and D_int_, respectively), and its further prolongation changes neither of those values. However, when the anodization time is fixed to 1 h, the outer layer starts to dissolve, revealing the nanotubular structure. Contrary to commonly applied multistep anodization of Ti, the change in applied potential does not result in the linear increase in the pore diameter for the one-step process. A slight decrease in surface parameters is observed for lower potentials, while for higher potentials the tendency is reversed. Such an unexpected behavior may be related to the nature of the initial oxide layer observed, i.e., the layer formed at the beginning of the anodization process, where different processes influence its geometry. The applied anodization conditions do not affect the elemental content of the formed layers nor the crystalline structure of the annealed samples, while they have an impact on the optical band-gap values. When the anodization potential varies between 40–60 V, the band-gap decreases for the amorphous samples. The decrease is further obtained by annealing the oxide layers, though the changes are potential-independent. The bang-gap reaches c.a. 3.05 eV, which is lower than that received for oxides formed on pure Ti.

To sum up, it was proven that the conditions of the one-step anodization process affect the surface morphology and properties of Ti-based alloys. It was also shown that due to the presence of the outer initial oxide layer, well-known dependencies between the applied anodization conditions and surface morphology parameters may not be accurate. Nonetheless, properties of anodized samples may be tuned by the process conditions, which gives almost limitless possibilities in designing new materials for various applications. Finally, it should be emphasized that this simple one-step anodization can be used for fast and effective generation of uniform oxide films on Ti15Mo without using time and energy-consuming multi-step processes.

## Figures and Tables

**Table 1:** An overview of oxidation conditions of TiMo alloys along with oxide layer morphology parameters. (D_p_—pore diameter, MSE - Mercury-mercurous Sulfate Electrode).

Alloy	Electrolyte	Type of the Process	Applied Potential and Time	Oxide Layer Morphology	Ref.
**Ti7Mo**	0.05 M HF in ethylene glycol (‘aged’ at 150 V for 20 h)	anodization in the three-electrode system (Pt and Ag/AgCl as counter and reference electrodes, respectively)	20–60 V 3 h	nanotubes with a nanoporous layer on the top D_p_ = 15–45 nm	[28]
**Ti7Mo**	0.18 M NH_4_F + 2 M H_2_O in ethylene glycol	anodization	50 V 1 h	nanotubes with a nanoporous layer on the top D_p_ = 100 nm	[29]
**Ti7.5Mo**	0.075 M NH_4_F in glycerol	anodization	20 V and 30 V 48 h	nanotubes D_p_ = 80 nm (20 V) D_p_ = 100 nm (30 V)	[18]
**Ti7.5Mo**	0.070 M NH_4_F + 0.55 M H_2_O in glycerol	anodization	20 V 24 h	nanotubes D_p_ = 65 nm	[17]
**Ti7.5Mo**	0.3 M NaCl with 0.14 M NH_4_F	anodization	10 V 60 min	nanotubes D_p_ = 31–44 nm	[30]
**Ti10Mo**	0.15 M NH_4_F + 1.25 M H_2_O in ethylene glycol	anodization	60 V 2 h	nanotubes with a porous and net film on the top of NT	[31]
**Ti15Mo**	0.5 M H_2_SO_4_; 1 M Na_2_SO_4_; 0.1 M NH_4_B_5_O_8_	potentiodynamic anodization	10–200 mV s^−1^ from the OCP value to 1–8 V vs. MSE	barrier-type anodic oxide with different oxide thickness	[20]
**Ti6Mo** **Ti15Mo**	0.25 M NH_4_F in ethylene glycol	anodization	20 or 60 V 6 h 40 V (2–6 h)	nanotubes D_p_ = 90 nm (for 40 V, 6 h) D_p_ = 100–120 nm (for 30 V)	[32]
**Ti15Mo**	0.075 M NH_4_F in glycerol	anodization	20 V 24 h	nanotubes D_p_ = ~65 nm	[33]
**Ti7Mo, Ti20Mo, Ti35Mo**	0.05 M HF in ethylene glycol (electrolyte was ‘aged’ at 150 V for 20 h)	anodization	50 V 35 min, 2 h or 3 h	Ti7Mo–nanotubes Ti20Mo and Ti35Mo–porous oxide layers	[34]
**Ti15Mo**	0.11 M NH_4_F + 1.11 M H_2_O in ethylene glycol	anodization	40–100 V 5–60 min	nanoporous oxide layers	this work

**Table 2:** Experimental conditions for the anodization of Ti15Mo samples in the ethylene glycol-based solution.

Potential (U)/V Time (t)/min	40	50	60	80	100
5	√				
10	√				
15	√	√	√	√	√
30	√				
60	√				

**Table 3:** Optical band gaps determined from UV–Vis diffuse reflectance spectra (DRS) for samples anodized at different potentials (both as-anodized and annealed).

U [V]	E_g_ [eV]	
	As-Received	Annealed
40	3.45 ± 0.05	3.05 ± 0.10
50	3.28 ± 0.06	3.09 ± 0.10
60	3.21 ± 0.03	3.08 ± 0.10
80	3.19 ± 0.04	2.95 ± 0.10
100	3.19 ± 0.02	3.04 ± 0.01

## Data Availability

Data sharing not applicable.

## Supplementary Materials

The following are available online at https://www.mdpi.com/2079-4991/11/1/68/s1, Figure S1: UV–Vis DRS spectra of TiMo samples anodized at 40 V (a) and 100 V (b) before (black lines) and after (red lines) thermal treatment in air at 400 °C for 2 h.
