# Supplementary material for: Electrochemical Oxidation of Ti15Mo Alloy—The Impact of Anodization Parameters on Surface Morphology of Nanostructured Oxide Layers
Source: Nanomaterials (Basel). 2020 Dec 30;11(1):68. doi: 10.3390/nano11010068 (PMC7824637; doi:10.3390/nano11010068)
Supplement: Supplementary file 1 [file nanomaterials-11-00068-s001.pdf]

SUPPLEMENTARY DATA

Electrochemical oxidation of Ti15Mo alloy – the impact of anodization parameters on surface morphology of nanostructured oxide layers

Magdalena Jarosz[1*], Leszek Zaraska[1*], Marcin Kozieł[1], Wojciech Simka[2], Grzegorz D. Sulka[1]

[1]*Jagiellonian University in Krakow, Faculty of Chemistry, Gronostajowa 2, 30387 Krakow, Poland*

[2]*Faculty of Chemistry, Silesian University of Technology, B. Krzywoustego 6, 44100 Gliwice, Poland*

Corresponding authors: jarosz@chemia.uj.edu.pl, zaraska@chemia.uj.edu.pl

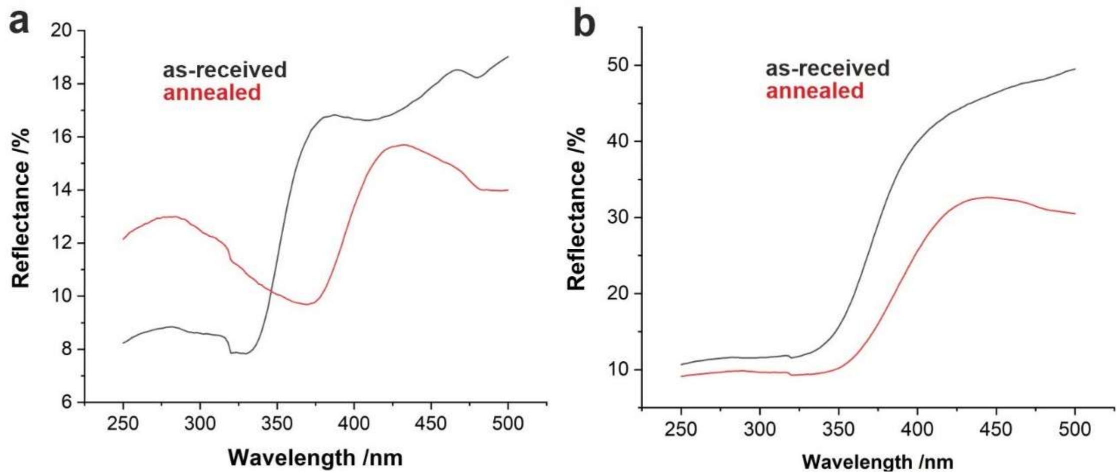

Figure S1. UV–Vis DRS spectra of TiMo samples anodized at 40 V (a) and 100 V (b) before (black lines) and after (red lines) thermal treatment in air at 400 °C for 2 h.
